# Effects of Novel Nitric Oxide-Releasing Molecules against Oxidative Stress on Retinal Pigmented Epithelial Cells

**DOI:** 10.1155/2017/1420892

**Published:** 2017-10-12

**Authors:** Valeria Pittalà, Annamaria Fidilio, Francesca Lazzara, Chiara Bianca Maria Platania, Loredana Salerno, Roberta Foresti, Filippo Drago, Claudio Bucolo

**Affiliations:** ^1^Department of Drug Sciences, University of Catania, Catania, Italy; ^2^Department of Biomedical and Biotechnological Sciences, Section of Pharmacology, School of Medicine, University of Catania, Catania, Italy; ^3^Inserm U955, Equipe 12, 94000 Créteil, France; ^4^Université Paris Est, Faculté de Médecine, 94000 Créteil, France; ^5^Center for Research in Ocular Pharmacology – CERFO, University of Catania, Catania, Italy

## Abstract

Oxidative stress is a hallmark of retinal degenerations such as age-related macular degeneration and diabetic retinopathy. Enhancement of heme oxygenase-1 (HO-1) activity in the retina would exert beneficial effects by protecting cells from oxidative stress, therefore promoting cell survival. Because a crosstalk exists between nitric oxide (NO) and HO-1 in promotion of cell survival under oxidative stress, we designed novel NO-releasing molecules also capable to induce HO-1. Starting from curcumin and caffeic acid phenethyl ester (CAPE), two known HO-1 inducers, the molecules were chemically modified by acylation with 4-bromo-butanoyl chloride and 2-chloro-propanoyl chloride, respectively, and then treated in the dark with AgNO_3_ to obtain the nitrate derivatives VP10/12 and VP10/39. Human retinal pigment epithelial cells (ARPE-19) subjected to H_2_O_2_-mediated oxidative stress were treated with the described NO-releasing compounds. VP10/39 showed significant (*p* < 0.05) antioxidant and protecting activity against oxidative damage, in comparison to VP10/12, which in turn showed at 100 *μ*M concentration a slight but significant cell toxicity. Only VP10/39 significantly (*p* < 0.05) induced HO-1 in ARPE-19, most likely through covalent bond formation at Cys151 of the Keap1-BTB domain, as revealed from molecular docking analysis. In conclusion, the present data indicate VP10/39 as a promising candidate to protect ARPE-19 cells against oxidative stress.

## 1. Introduction

Oxidative stress plays a key role in the pathogenesis of several eye disorders, such as, age-related macular degeneration (AMD) and diabetic retinopathy (DR) [1–5]. Heme oxygenase-1 (HO-1), also known as heat shock protein 32 (HSP32), is one of the components of cellular defense mechanisms against oxidative stress-mediated injury. The transcription of HO-1 is mediated by the nuclear transcription factor Nrf2, whose protein levels are in turn regulated by the Kelch-like ECH-associated protein 1 (Keap1) [6].

Regarding AMD, the role of HO-1 in disease pathogenesis has been highlighted by genetic association of the 19G>C HO-1 gene variant with incidence and progression of AMD. Furthermore, the gene variant 25129A>C of Nfr2, activator of HO-1 expression, was associated to AMD. Nfr2^−/−^ mice developed age-dependent degeneration of the RPE and choriocapillaris function, along with spontaneous choroidal neovascularization and deposits of inflammatory proteins in the subretinal space [7]. In this perspective, activation of the Nrf2-HO1 axis would be beneficial for the treatment of AMD.

The inducible HO isoform, HO-1, is highly expressed in the retina of diabetic rats [8], and increased levels of HO-1 may be a response to oxidative stress in diabetes [9, 10]. However, it was found that long-term diabetes led to reduced HO-1 mRNA levels in the retinal pigmented epithelium (RPE) [11]. Therefore, induction of HO-1 would be beneficial also for the treatment of diabetic retinopathy.

Curcumin and caffeic acid phenethyl ester (CAPE) are nutraceutical substances able to induce the upregulation of HO-1. Studies have shown that curcumin has a wide range of beneficial properties, including anti-inflammatory and antioxidant activities [12, 13]. The pleiotropic action of curcumin is related to regulation of multiple survival and cytoprotective signaling pathways, including anti-inflammatory pathways and those regulated by NF*κ*B, AKT, growth factors, and Nrf2 transcription factor [14–23].

Specifically, low concentrations of curcumin induced HO-1 expression in RPE cells and decreased reactive oxygen species (ROS) in RPE cells, challenged with hydrogen peroxide [24]. Additionally, curcumin modulated retinal oxidative stress in a rat model of streptozotocin- (STZ-) induced diabetic retinopathy [25]. CAPE exhibited antioxidant [26] and anti-inflammatory properties [27] that can be exploited for treatment of several conditions such as ischemia/reperfusion injury [28, 29], atherosclerosis [30] and diabetes [31]. The cytoprotective effect of CAPE against oxidant stress is due to upregulation of HO-1 mRNA [32], through induction of transcription of the ARE-related (antioxidant response element) gene [33]. The mechanism of action of CAPE was confirmed in several *in vitro* studies [32, 34], showing that CAPE significantly increase HO-1 protein expression through inactivation of Nrf2-Keap1 complex and consequent Nrf2 activation [22].

Paeng et al. [35] showed that CAPE inhibits VEGF production in an *in vitro* model of retinal hypoxia (ARPE-19 cells under hypoxic conditions). Thus, CAPE would reduce retinal neovascularization through inhibition of ROS synthesis and reduction of HIF-1*α* and VEGF expression [35].

Another molecule that influences the regulation of HO-1 production is nitric oxide (NO) [36]. This diatomic molecule is recognized as an important intercellular messenger in biological systems, for example, the cardiovascular system and the nervous system, including the retina. NO is synthesized by NO synthase isoforms, expressed by endothelial cells and efferent nitrergic neurons. NO is an important modulator of homeostatic processes in the eye, such as regulation of aqueous humor dynamics, blood flow, retinal neurotransmission, and phototransduction. An imbalance in NO production is associated with pathological states such as inflammatory diseases (uveitis, retinitis) or degenerative diseases (glaucoma, retinal degeneration) [37]. The reduction of the bioavailability of NO, caused, for example, by endothelial dysfunction, increases the production of ROS. However, overproduction of NO could be harmful, such as the sustained NO synthesis by inducible NOS (iNOS), which is expressed in response to an inflammatory event. Therefore, it is difficult to design an effective therapeutic strategy with NO supplementation or NOS inhibition, due to the dual action of NO [38]. However, latanoprostene bunod, a NO-releasing prostaglandin analog, is expected to be approved by FDA in the near future for clinical use in glaucoma [39].

The HO and NOS systems show numerous and several interactions. For instance, both are activated by ROS and cytokines [40] and can activate guanylyl cyclase [41]. On the other hand, NO can upregulate HO expression by means of a cGMP-dependent pathway [42]. Datta et al. demonstrated that NO induces HO-1 and showed that there are interactions between the iNOS and HO-1 pathways [43]. Furthermore, Chen and Maines demonstrated that exposure of HeLa cells to the NO donor, sodium nitroprusside (SNP), induced a concentration and time-dependent increase of HO-1 mRNA and activation of mitogen-activated protein kinases (MAPKs): the ERK (ERK1 and ERK2) and p38 pathways [44].

**Figure 1:**
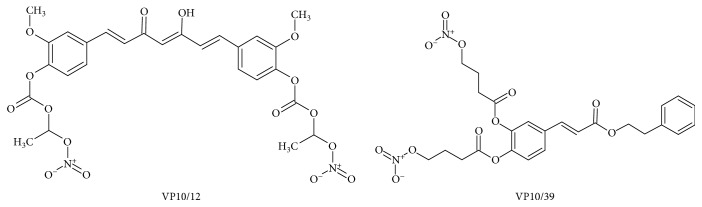
Chemical structure of the synthesized NO-releasing caffeic acid phenethyl ester (VP10/39) and NO-releasing curcumin (VP10/12).

In this perspective, we designed and synthesized the novel NO-releasing molecules VP10/12 and VP10/39, bearing curcumin and CAPE scaffolds, respectively (Figure 1()). These compounds decreased significantly oxidative stress and increased cell viability of ARPE-19 challenged with hydrogen peroxide. VP10/39, the CAPE-NO derivative, was more effective than VP10/12, most likely due to its capability to induce HO-1 in ARPE-19 cells.

## 2. Materials and Methods

### 2.1. Chemistry, Drugs, Chemicals, and Reagents

Melting points of newly synthesized derivatives were determined with an Electrothermal IA9200 using glass capillary tubes. Infrared spectra were recorded on a Perkin–Elmer FT IR 1600 spectrometer in KBr disks. Elemental analyses for C, H, and N were within 70.4% of theoretical values and were performed on a Carlo Erba Elemental Analyzer Mod. 1108 apparatus. ^1^H NMR spectra were recorded at 200 MHz on a Varian Inova Unity 200 spectrometer in DMSO-*d*_6_ or chloroform-*d* solution. Chemical shifts are given in *δ* values (ppm), using tetramethylsilane as the internal standard; coupling constants (*J*) are given in hertz (Hz). Signal multiplicities are characterized as s (singlet), d (doublet), t (triplet), q (quartet), m (multiplet), and br (broad signal). All the synthesized compounds were tested for purity on TLC (aluminum sheet coated with silica gel F254, Merck) and visualized by UV (*λ* 254 and 366 nm).

#### 2.1.1. Synthesis of bis-4-[4-(Nitrooxy)-1-Butoxy]-Caffeic Acid Phenethyl Ester (VP10/39)

**Figure 2:**
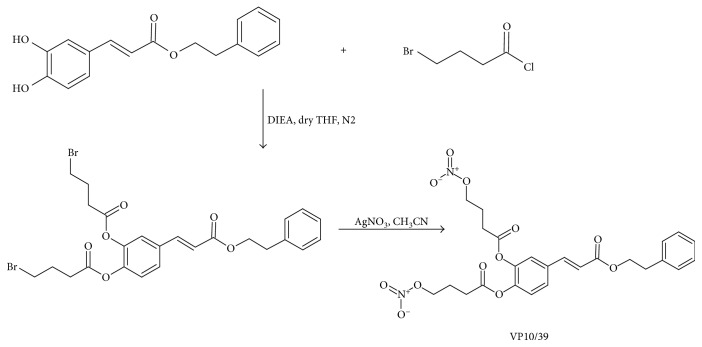
Synthesis of NO-releasing CAPE (VP10/39).

A solution of 4-bromo-butanoyl chloride (2.5 mmol) and *N*,*N*-diisopropylethylamine (2.5 mmol) in 1 mL of anhydrous THF was slowly added to a solution of CAPE (1 mmol) in 5 mL of anhydrous THF, cooled at 0°C, and under a nitrogen atmosphere. Following the addition, the reaction mixture was left stirring at room temperature for 1 h. The obtained solution was diluted with 70 mL of EtOAc and the organic layer was washed with water (2 × 50 mL) and brine (1 × 50 mL), dried over anhydrous Na_2_SO_4_, filtered, and evaporated. The crude material was dissolved in 7 mL of CH_3_CN, 2.5 mmol of AgNO_3_ was added, and the mixture was left stirring at reflux for 3 h, in the dark. The obtained suspension was filtered and evaporated (Figure 2()). The residue was purified by flash chromatography on silica gel 60 using a mixture of cyclohexane/EtOAc (7/3, *v*/*v*) as an eluent. Homogeneous fractions were combined and evaporated under reduced pressure to give the title compound (0.38 g, yield 70%) as a pure light-yellow oil: IR (KBr) cm^−^^1^ 2959, 1770, 1712, 1635, 1505, 1280, 1172, 1112, 869; ^1^H NMR (DMSO-*d*_6_): *δ* 7.79–7.57 (m, 2H + 1H, aromatic + C*H* = CHCOO), 7.39–7.18 (m, 6H, aromatic), 6.63 (d, *J* = 16 Hz, 1H, CH = C*H*COO), 4.64 (t, *J* = 6.4 Hz, 2H, CH_2_CH_2_C*H*_2_ONO_2_), 4.60 (t, *J* = 6.4 Hz, 2H, CH_2_CH_2_C*H*_2_ONO_2_),4.37 (t, *J* = 6.8 Hz, 2H, COOC*H*_2_CH_2_), 2.97 (t, *J* = 6.8 Hz, 2H, COOCH_2_C*H*_2_), 2.80–2.67 (m, 2H + 2H, C*H*_2_CH_2_CH_2_ONO_2_), 2.12–1.94 (m, 2H + 2H, CH_2_C*H*_2_CH_2_ONO_2_). Anal. (C_25_H_26_N_2_O_12_) C, H, N.

#### 2.1.2. Synthesis of [(1E,6E)-3,5-Dioxohepta-1,6-Diene-1,7-Diyl]bis-2-Methoxy-4,1-Phenylene bis[1-(Nitrooxy)Ethyl] Biscarbonate (VP10/12)

**Figure 3:**
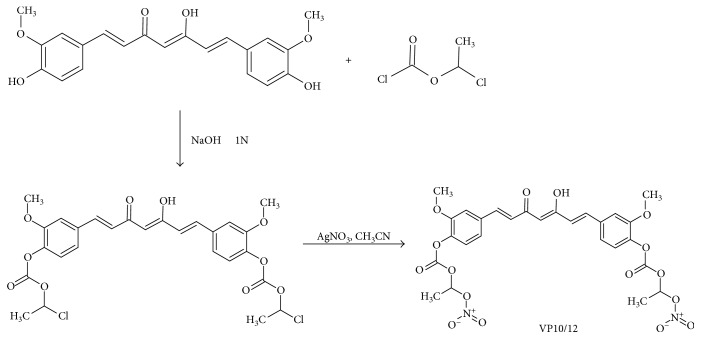
Synthesis of NO-releasing curcumin (VP10/12).

(1*E*,6*E*)-1,7-bis(4-[[[1-(chloro)ethoxy]carbonyl]oxy]-3-methoxyphenyl)hepta-1,6-diene-3,5-dione (0.5 mmol) was dissolved in 3 mL of CH_3_CN; 1.0 mmol of AgNO_3_ was added; and the mixture was left stirring at reflux for 3 h, in the dark (Figure 3()). The obtained suspension was filtered and evaporated. The residue was purified by flash chromatography on silica gel 60 using a mixture of cyclohexane/EtOAc (7/3, *v*/*v*) as an eluent. Homogeneous fractions were combined and evaporated under reduced pressure to give the title compound (0.20 g, yield 62%) as a pure yellow-orange oil: IR (KBr) cm^−^^1^ 2953, 2929, 2880, 1635, 1621, 1510, 1438, 1257, 1027, 870; ^1^H NMR (CDCl_3_): *δ* 7.62 (d, *J* = 16 Hz, 1H + 1H, ArCH = C*H*), 7.27–7.10 (m, 6H, aromatic), 7.02 (q, *J* = 5.6 Hz, 1H + 1H, C*H*CH_3_), 6.58 (d, *J* = 16 Hz, 1H + 1H, ArC*H* = CH), 5.87 (s, 1H, COC*H* = COH), 3.91 (s, 3H + 3H, OCH_3_), 1.68 (d, *J* = 5.6 Hz, 3H + 3H, CHC*H*_3_). Anal. (C_27_H_26_N_2_O_16_) C, H, N.

#### 2.1.3. Synthesis of bis(1-Chloroethyl)[(1E,6E)-3,5-Dioxohepta-1,6-Diene-1,7-Diyl]bis-2-Methoxy-4,1-Phenylene Biscarbonate

Curcumin (2 mmol) was dissolved in 20 mL of ice-cooled NaOH 1N, and 2-chloro-propanoyl chloride (8 mmol) was added (Figure 3()). Following the addition, the reaction mixture was left stirring at room temperature for 15 min and filtered. The obtained crude material was purified by flash chromatography on silica gel 60 using a mixture of cyclohexane/EtOAc (5/5, *v*/*v*) as an eluent. Homogeneous fractions were combined and evaporated under reduced pressure to give the title compound (0.32 g, yield 28%) as a pure yellow solid: mp 104-105°C. IR (KBr) cm^−^^1^ 3003, 2955, 2870, 1620, 1597, 1581, 1508, 1422, 1250, 1120, 770 ^1^H NMR (CDCl_3_): *δ* 7.70 (d, *J* = 16 Hz, 1H + 1H, ArCH = C*H*), 7.38–7.20 (m, 6H, aromatic), 6.37–7.19 (m, 1H + 1H + 1H + 1H, ArC*H* = CH + C*H*CH_3_), 5.95 (s, 1H, COC*H* = COH), 3.99 (s, 3H + 3H, OCH_3_), 2.00 (d, *J* = 6 Hz, 3H + 3H, CHC*H*_3_). Anal. (C_27_H_26_Cl_2_O_10_) C, H.

### 2.2. *In Vitro* Studies

ARPE-19 (human retinal pigment epithelial) cells were purchased from ATCC®. The cell line was cultured at 37°C (humidified atmosphere with 5% CO2), in ATCC-formulated DMEM:F12 medium (ATCC number 30-2006) with 100 U/mL penicillin, 100 *μ*g/mL streptomycin, and 10% fetal bovine serum.

ROS were measured with the DCFDA—Cellular Reactive Oxygen Species Detection Assay Kit (ab113851)—according to manufacturer's protocol. DCFDA, a cell permeable fluorogenic dye, is deacetylated by cellular esterases to a nonfluorescent compound and later oxidized by ROS into highly fluorescent 2′,7′-dichlorofluorescein (DCF); fluorescence intensity is proportional to cell ROS concentration. ARPE-19 cells were plated into 96-well black plates (2 × 10*E*^4^ cells per well), and confluence was reached within 24 h. After confluence, cells were washed twice with phosphate-buffered saline (PBS pH 7.4) and incubated with 25 *μ*M DCFDA in buffer solution (provided with the kit) at 37°C for 45 minutes. After two washes with PBS, cells were treated with increasing concentrations of VP10/39 and VP10/12 (1–10–20–100 *μ*M) for 60 minutes, then oxidative stress was induced with 500 *μ*M H_2_O_2_ treatment for 120 minutes. ROS concentration was measured by detection of DCF fluorescence (*λ*_ex_ = 495 nm, *λ*_em_ = 529 nm) with a Varioskan™ Flash Multimode Reader.

Lactate dehydrogenase (LDH) cell release was measured using the Cytotoxicity Detection KitPLUS (LDH) (ROCHE 04744934001) according to manufacturer's protocol. LDH is released into the medium when the integrity of the cell membrane is lost; therefore, high levels of LDH are indices of cell death. ARPE-19 cells were plated into 96-well plates (1.5 × 10*E*^4^ cells per well). After cell confluence was reached (24 h), cells were treated with increasing concentrations of VP10/39 and VP10/12 (1–10–20–100 *μ*M) for 60 minutes and cell damage was induced by 2 mM H_2_O_2_ for 60 minutes. Cell-free culture supernatant was collected in empty plates and incubated with the enzymatic reaction mixture for 30 minutes. LDH was quantified by measuring absorbance at 490 nm with the Varioskan Flash Multimode Reader.

ATP is present in metabolically active cells and its concentration declines when cell necrosis and apoptosis occur. After oxidative stress induction by treatment with H_2_O_2_, ATP production was measured using the ATPlite 1 step kit (Perkin Elmer 6016731), according to manufacturer's protocol. ATP concentration is proportional to luminescence intensity related to ATP reaction with luciferase and D-luciferin. ARPE-19 cells were plated into 96-well white plates (1.5 × 10*E*^4^ cells per well). Cells were treated with increased concentrations of VP10/39 and VP10/12 (1–10–20–100 *μ*M) for 60 minutes, then oxidative stress was induced with 1.5 mM H_2_O_2_ for 180 minutes. After that, the plate was equilibrated at room temperature (20–22°C), before addition of the reaction solution; therefore, luminescence was measured with Varioskan Flash Multimode Reader.

### 2.3. Heme Oxygenase Activity Assay

ARPE-19 cells were cultured in 100 mm diameter petri dishes and were collected 6 hours after incubation with VP10/12 and VP10/39 at 10 *μ*M and 20 *μ*M concentrations. The heme oxygenase activity assay was then carried out on the basis of the spectrophotometric determination of bilirubin as the final product of heme degradation by heme oxygenase as described previously by Foresti et al. [45]. Collected cells were incubated with hemin, NADPH, and liver cytosol (a source of biliverdin reductase); the reaction was carried out at 37°C in the dark and was stopped after 1 h with an addition of chloroform, used in order to extract the produced bilirubin. Heme oxygenase activity is expressed as picomoles of bilirubin/mg protein/60 min.

### 2.4. Statistical Analysis

All results were reported as mean ± SD. Statistical analysis was carried out using 1-way ANOVA followed by Tukey-Kramer multiple comparison test. Differences between two groups were considered as significant given a *p* value < 0.05. Graphs were done using GraphPad Prism 5 software (GraphPad Inc., San Diego, CA) that was also used for statistical analysis.

### 2.5. Molecular Modeling

Activation of the Nrf2 pathway occurs with inhibition of the KEAP1/Nrf2 protein-protein interaction or Keap1 dimerization. Therefore, Nrf2 inducers act by

- disrupting the Keap1-DC domain/Nfr2 interaction [46];
- covalent binding to cysteine residues of the Keap1-BTB domain [47].

We investigated the binding of VP10/12 and VP10/39 at Keap1-DC and Keap1-BTB with a molecular modeling approach. The whole human Keap1 dimer was built with the advanced molecular modeling task of Maestro© and subjected to loop optimization and two energy minimization steps: rigid body energy and all-atom energy minimization using the VSGB 2.0 solvation model [48]. BTB and IVR domains of Keap1 were modeled on the basis of BTB and BACK (IVR) domains of Keltch11 (PDB:3I3N); the DC domain of Keap1 was modeled using the PDB:1X2R X-ray structure as template. The quality of the model was assessed by determination of protein Ramachandran plots before and after energy minimization; the two energy minimization steps led to a significant decrease of residue dihedral violations (Figure 1S supplemental material available online at https://doi.org/10.1155/2017/1420892). Superimposition between the model of human Keap1 dimer with the electron microscopy reconstruction map (24 Å) of murine Keap1 dimer [49] was carried out with Chimera 1.11.2 (Figure 2S supplemental material). RMSD between human Keap1 dimer model and electron microscopy map of murine Keap1 was 4.5 Å. VP10/12 and VP10/39.sdf files were built with the web server “Online SMILES Translator and Structure File Generator” https://cactus.nci.nih.gov/translate/. Tridimensional structures of these two ligands, tautomerization and ionization at pH 7.4, were obtained by launching the LigPrep task of Schrodinger© Maestro. Molecular docking of curcumin and CAPE was carried out in order to compare binding of novel synthetized compounds with other known Nrf2 inducers. We used the following docking protocol on the Keap1-DC domain [46]: (i) grid generation on the centroid of the binding pocket; (ii) standard precision (SP) docking (Schrodinger©) performed with Glide (Schrodinger©). At first, docking was carried out with ring conformation sampling, followed by a 500-step conjugate gradient minimization by Glide (dielectric = 1). Covalent docking of VP10/12 and VP10/39 was carried out at the BTB domain of Keap1 as follows: Cys 151 was set as reactive residue and the Michael's addition reaction was simulated [47] with CovDock task of Schrodinger© Maestro [50]. MMGBSA rescoring of semiflexible docking at the DC domain and covalent docking at BTB was carried out with Schrodinger© Maestro: the VSGB 2.0 model was used and all residues within 10 Å from ligand were allowed to move.

## 3. Results

### 3.1. Chemical Synthesis of NO-Releasing Curcumin (VP10/12) and NO-Releasing CAPE (VP10/39)

Hybrid NO-releasing molecules were obtained in high yield by using an efficient synthetic route (Figures 2() and 3()). The alcoholic-free groups of CAPE and curcumin were firstly acylated with 4-bromo-butanoyl chloride or 2-chloro-propanoyl chloride, respectively. In a subsequent step, the intermediates were converted into the corresponding nitrate derivatives by the treatment with AgNO_3_ at reflux into the dark. The desired final products, VP10/12 and VP10/39, were easily obtained by flash chromatography purification. VP10/12 was obtained in a high overall yield, 70% two steps; on the contrary, VP10/39 was obtained in poor yield because it is easily hydrolyzed under purification conditions.

### 3.2. VP10/12 and VP10/39 Protected ARPE-19 Cells from Oxidative Stress

**Figure 4:**
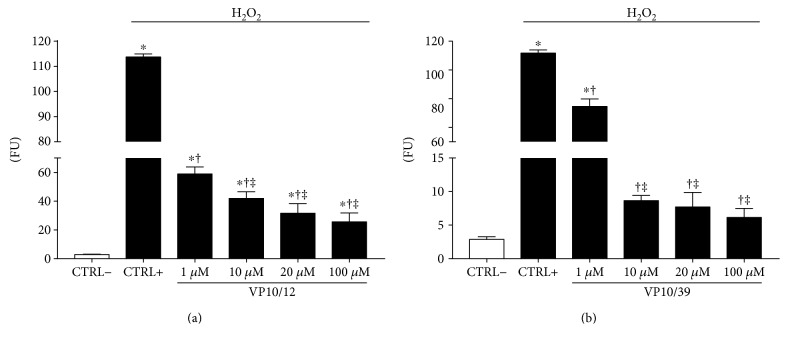
VP10/12 and VP10/39 decreased ROS concentration in ARPE-19 cells challenged with H_2_O_2_. ^∗^*p* < 0.05 versus CTRL− cells (cells that were not treated with H_2_O_2_), ^†^*p* < 0.05 versus CTRL+ cells (cells that were treated with H_2_O_2_), ^‡^*p* < 0.05 versus 1 *μ*M VP10/12 or VP10/39 treatment.

We tested the antioxidant properties of VP10/12 and VP10/39 in ARPE-19 cells, challenged with H_2_O_2_. Treatment with H_2_O_2_ induced oxidative stress by increasing ROS concentration ([ROS]) in ARPE-19 cells (Figures 4(a) and 4(b)). While, pretreatment (60 min) with VP10/12 and VP10/39 decreased significantly (*p* < 0.05) [ROS] in ARPE-19 subjected to oxidative stress (Figures 4(a) and 4(b)). The CAPE-NO derivative, VP10/39, at 1 *μ*M concentration induced a 1.5-fold decrease of [ROS]. Furthermore, 10 *μ*M VP10/39 decreased ROS concentration to values of negative control cells (CTRL−), 8.7 ± 0.8 FU and 2.9 ± 0.4 FU, respectively (FU = fluorescent units, Figure 4(b)). The curcumin-NO derivative VP10/12 induced at 1 *μ*M concentration a significant 2-fold decrease of [ROS] in ARPE-19 cells treated with H_2_O_2_. Additionally, VP10/12 inhibited ROS formation in a dose-dependent manner. VP10/12 did not decrease [ROS] to levels of negative control cells, in contrast to data obtained for VP10/39.

**Figure 5:**
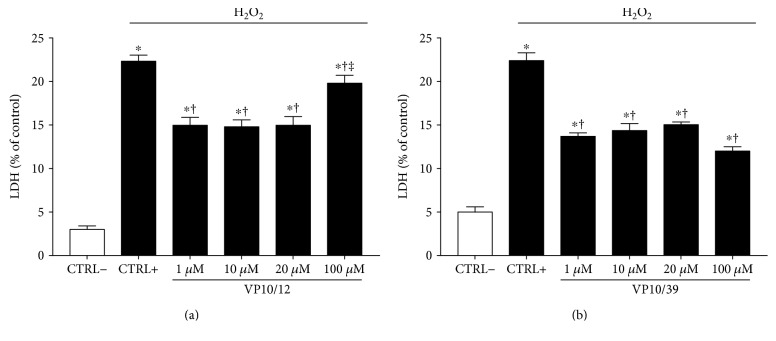
VP10/12 and VP10/39 decreased LDH release from ARPE-19 cells challenged with H_2_O_2_. ^∗^*p* < 0.05 versus CTRL− cells (cells that were not treated with H_2_O_2_), ^†^*p* < 0.05 versus CTRL+ cells (cells that were treated with H_2_O_2_), ^‡^*p* < 0.05 versus 1 *μ*M VP10/12 or VP10/39 treatment.

We analyzed the LDH release in medium of ARPE-19 challenged with H_2_O_2_, in order to assess the protective effects of tested compounds on cell death induced by oxidative stress. VP10/12 and VP10/39 significantly (*p* < 0.05) induced a 1.5-fold decrease in LDH release, in comparison to positive control cells (CTRL+, ARPE-19 treated with H_2_O_2_, Figures 5(a) and 5(b)). However, 100 *μ*M VP10/12 increased LDH levels (19.80 ± 0.90) to values reported for CTRL+ cells (22.33 ± 0.70); thus curcumin-NO might exert toxic effects at high concentrations.

**Figure 6:**
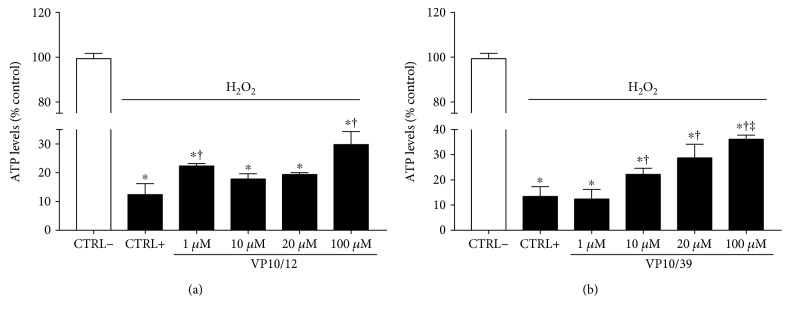
VP10/12 and VP10/39 viability of ARPE-19 cells challenged with H_2_O_2_. ^∗^*p* < 0.05 versus CTRL− cells (cells that were not treated with H_2_O_2_), ^†^*p* < 0.05 versus CTRL+ cells (cells that were treated with H_2_O_2_), ^‡^*p* < 0.05 versus 1 *μ*M VP10/12 or VP10/39 treatment.

Oxidative damage affected cellular viability of ARPE-19 cells, given a significant (*p* < 0.05) decrease of ATP levels in CTRL+ cells, in comparison to CTRL− cells (Figures 6(a) and 6(b)). Treatment with VP10/12 and VP10/39 increased significantly ATP levels in ARPE-19 cells challenged with H_2_O_2_ (Figures 6(a) and 6(b)). Interestingly, VP10/39 treatment increased ARPE-19 cell viability in a dose-dependent manner.

Overall, these data indicated that both VP10/12 and VP10/39 are capable to protect ARPE-19 cells from oxidative damage; however, VP10/39 was more effective in decreasing ROS formation and LDH release than VP10/12, which at highest concentration (100 *μ*M) exerted a slight, still significant, toxic effect on ARPE-19 cells.

### 3.3. VP10/39 Induced HO-1

**Figure 7:**
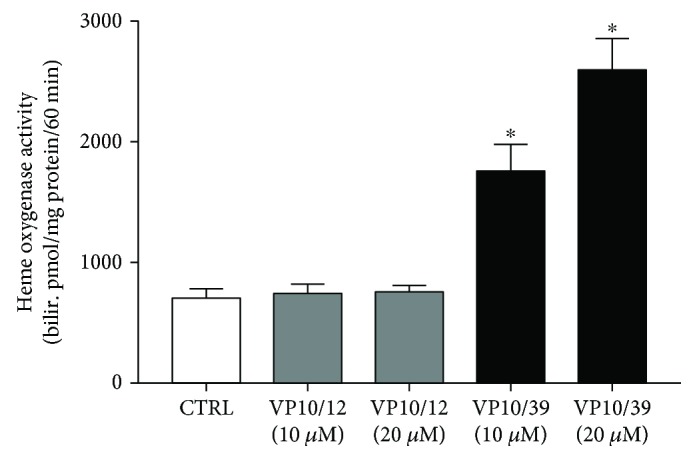
HO-1 induction by VP10/12 and VP10/39. ^∗^*p* < 0.05 versus CTRL control cells.

Resistance to oxidative stress of cells treated with VP10/12 and VP10/39 can be accounted not only to ROS-scavenging capability but also to induction of HO-1. We tested the capability of VP10/12 and VP10/39 to induce HO-1 in ARPE-19 by means of a HO-1 activity assay, which was previously set for ARPE-19 cells [45]. Only VP10/39 induced significantly (*p* < 0.05) HO-1 in ARPE-19 at both tested concentrations, 10 *μ*M and 20 *μ*M. VP10/12 at 10 *μ*M and 20 *μ*M concentrations did not alter HO-1 activity in ARPE-19 cells, because no substantial differences were found between control and VP10/12-treated cells (Figure 7()). This result would explain the significant greater antioxidant activity of VP10/39, compared to VP10/12 (Figure 4()).

**Figure 8:**
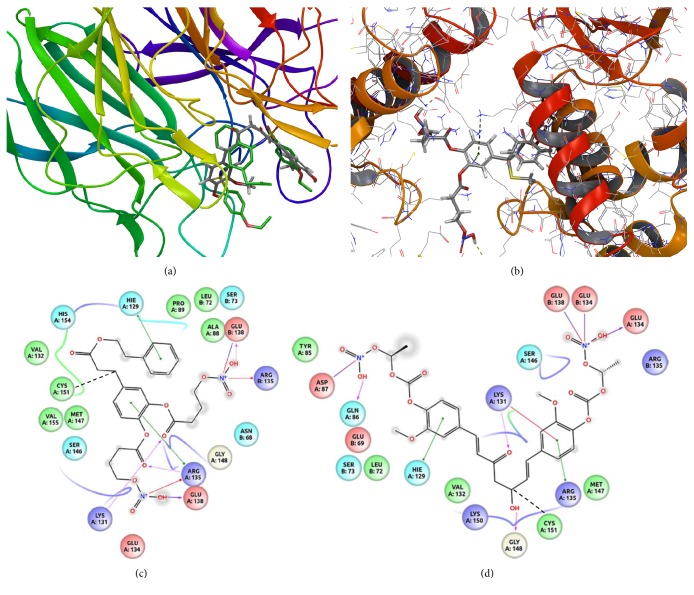
Molecular docking into Keap1 domains. (a) Semiflexible docking at the Keap1-DC domain of curcumin (grey stick) and K67 (PDB:4ZY3, green stick). (b) Covalent docking—Michael addition at the Cys151 of the BTB domain of Keap1. (c) 2D pose representation of VP10/39 covalent bound (black hatched line) to Cys151 of the Keap1-BTB domain. (d) 2D pose representation of VP10/12 covalent bound (black hatched line) to Cys151 of the Keap1-BTB domain.

In order to rationalize the capability of VP10/39 to induce HO-1, we built the model of Keap1 dimer, which, through association in a multiprotein complex, regulates the degradation of the nuclear factor Nrf2. In fact, under oxidative condition, Keap1 dimer dissociates and is not able to keep Nrf2 immobilized for ubiquitination by the E2 ubiquitin [51]. HO-1 induction can be promoted by stabilization of Nrf2 protein levels, therefore, by either inhibiting interaction between Keap1-DC/Nrf2 [46] or destabilizing the Keap1 dimer at the BTB domain [47]. In this perspective, we carried out semiflexible docking of VP10/12, VP10/39, curcumin, and CAPE at DC. Furthermore, we simulated Michael addition reaction (covalent docking) of ligands at the dimeric BTB domain of Keap1. Semiflexible docking of VP10/12 and VP10/39 at the DC domain of Keap1 was characterized by better docking scores (more negative) in comparison to both curcumin and CAPE. VP10/12 showed a slightly better docking score at the DC domain (Glide score = −7.66), if compared to VP10/39 (Glide score = −7.22). However, poses (binding modes) of VP10/12 and VP10/39 are not similar to poses of known ligands of Keap1-DC. This trend was confirmed by MMGBSA rescoring (Table 1). On the contrary, curcumin (Glide score = −5.74) complexed at Keap1-DC alike a crystallized Keap1-DC/ligand complex (PDB:4ZY3) [46] (Figure 8(a)). Docking score differences between VP10/12 and VP10/39 at the Keap1 DC domain did not explain the capability of VP10/39 to induce HO-1. Worthy of note, MMGBSA binding energy was more negative for binding of ligands at the DC domain than binding of ligands at the BTB domain. However, MMGBSA rescoring for docking at DC and BTB cannot be compared, because of significant differences in simulated complex formation: noncovalent binding versus covalent binding. Covalent docking of the tested compounds at the Keap1-BTB domain revealed that VP10/39 covalently bound to Cys 151 of Keap1-BTB showed better affinity (−7.06) than VP10/12 (−5.63), curcumin (−4.07), and CAPE (−3.33) (Figures 8(b), 8(c), and 8(d)). MMGBSA covalent docking rescoring confirmed that VP10/39 bound covalently to BTB domain with the lowest binding-free energy than other ligands. Indeed, VP10/39 can induce HO-1 by means of a covalent bound to Cys151 of Keap1-BTB, inhibiting Keap1 dimerization at the BTB domain.

## 4. Discussion

This study was aimed at designing new NO-releasing molecules to be potentially used for the treatment of AMD and DR. Oxidative stress, along with inflammation, is a hallmark of age-related retinal diseases such as AMD and DR, that lead to irreversible sight loss, if not correctly managed [52, 53]. Currently, there are no approved drugs for the treatment of early phases of AMD and DR. Besides that, antiangiogenic and anti-inflammatory drugs are approved for treatment of diabetic macular edema and wet form of AMD, which are clinical forms of AMD and DR progression. Dysregulation of the HO-1 defense system against oxidative cell damage has been linked to AMD and DR progression. Antioxidant supplementation has been explored as a therapeutic strategy for diabetic retinopathy (clinicaltrial.gov “antioxidant AND diabetic retinopathy”) and AMD (clinicaltrial.gov “antioxidant AND age related macular degeneration”). Furthermore, there are clinical trials about improvement of visual function in patients with diabetic retinopathy treated with curcumin supplementation (NCT02984813, NCT01646047). Besides preclinical promising results [24, 25, 35], no clinical studies have been reported about either curcumin as a treatment of AMD or CAPE as treatment of both DR and AMD. We hereby hypothesized that a HO-1 inducer with a NO-releasing moiety would be promising for protection of retinal pigmented epithelium against oxidative stress, because NO can also induce HO-1. Both VP10/12 (curcumin-NO-releasing derivative) and VP10/39 (CAPE-NO-releasing derivative) were able to decrease ROS concentration and LDH release. Furthermore, both compounds increased cell viability in ARPE-19 cells, challenged with H_2_O_2_. However, VP10/39 was more effective in decreasing ROS and LDH in retinal pigmented epithelial cells treated with H_2_O_2_, in comparison to VP10/12.

Moreover, VP10/12 showed significant cell toxicity effects (*p* < 0.05) at the highest tested concentration (100 *μ*M); this result can be related either to a toxic dose of curcumin from hydrolyzed VP10/12 or to rapid and sustained release of NO by VP10/12.

The better activity of VP10/39, in comparison to VP10/12, can be explained by its capability to induce HO-1, as well as other CAPE derivatives [54], likely by means of covalent binding to Cys 151 of the Keap1-BTB domain, which is involved in the dimerization of Keap1. In fact, dimerization of Keap1 is a necessary condition for ubiquitination and degradation of Nrf2; therefore, oligomerization of Keap1 is a good strategy for stabilizing Nrf2 and consequently to induce HO-1 [47].

## 5. Conclusions

In conclusion, VP10/39 is a promising compound capable of protective activity against oxidative damage on retinal pigmented epithelium. Therefore, this compound could be further developed as adjuvant antioxidant treatment of AMD and diabetic retinopathy.

## Supplementary Material

Supplemental figures show the results (Ramachandran Plots) of energy minimization of human Keap1 dimer model (Fig 1S) and the superimposition of the full atom model with the low resolution (24 Å) structure of murine Keap1 dimer (Fig 2S). Fig. 1 S. Ramachandran-plots of Keap-1 dimer. A) Plot before energy minimization. B) Plot after energy minimization. Energy minimization (rigid body and all-atom) was carried out with the protein optimization task of Schrodinger © Maestro using the VSGB 2.0 solvation model. Fig.2S. Superimposition of Keap1 dimer model with electron microscopy reconstruction map of mice Keap1 dimer. Mesh surface corresponds to volumetric electron microscopy reconstruction map (24 Å) of murine Keap1 dimer, magenta cartoon corresponds to all-atom model of human Keap1 dimer obtained with Schrodinger © Maestro Advanced Homology modeling task.

## Figures and Tables

**Table 1:** Docking scores, covalent affinity, and Δ*G*_binding_ of docked ligands. Glide scores and covalent affinities are reported as arbitrary units; Δ*G*_binding_ are expressed in Kcal/mol.

Ligand	Semiflexible docking at DC domain		Covalent docking at BTB domain	
	Glide score	Δ*G*_binding_	Covalent affinity	Δ*G*_binding_
VP10/12	−7.66	−90.05	−5.63	−50.42
VP10/39	−7.22	−86.70	−7.06	−68.04
Curcumin	−5.74	−67.50	−4.07	−47.38
CAPE	−4.08	−62.50	−3.33	−41.67
