# Supplementary material for: Effects of Novel Nitric Oxide-Releasing Molecules against Oxidative Stress on Retinal Pigmented Epithelial Cells
Source: Oxid Med Cell Longev. 2017 Oct 12;2017:1420892. doi: 10.1155/2017/1420892 (PMC5660806; doi:10.1155/2017/1420892)

**Supplemental material**

Supplemental figures show the results (Ramachandran Plots) of energy minimization of human Keap1 dimer model (Fig 1S) and the superimposition of the full atom model with the low resolution (24 Å) structure of murine Keap1 dimer (Fig 2S).

**Fig. 1 S. Ramachandran-plots of Keap-1 dimer.** A) Plot before energy minimization. B) Plot after energy minimization. Energy minimization (rigid body and all-atom) was carried out with the protein optimization task of Schrodinger © Maestro using the VSGB 2.0 solvation model.


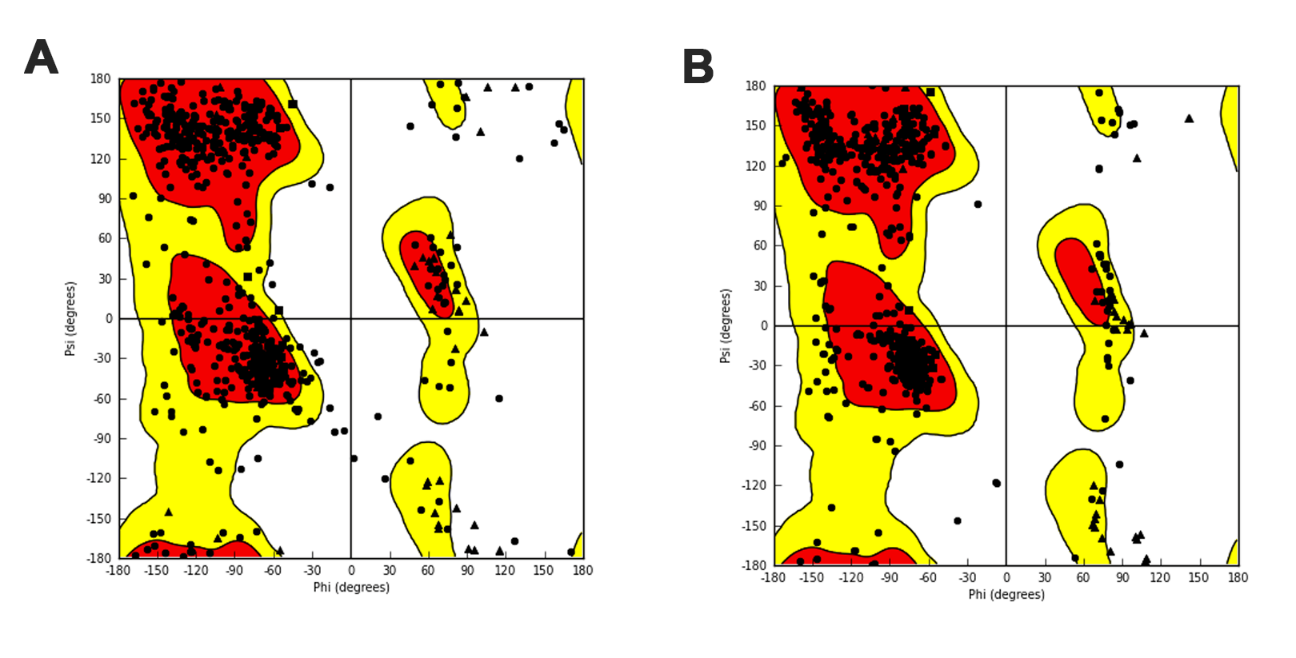


**Fig.2S. Superimposition of Keap1 dimer model with electron microscopy reconstruction map of mice Keap1 dimer.** Mesh surface corresponds to volumetric electron microscopy reconstruction map (24 Å) of murine Keap1 dimer, magenta cartoon corresponds to all-atom model of human Keap1 dimer obtained with Schrodinger © Maestro Advanced Homology modeling task.


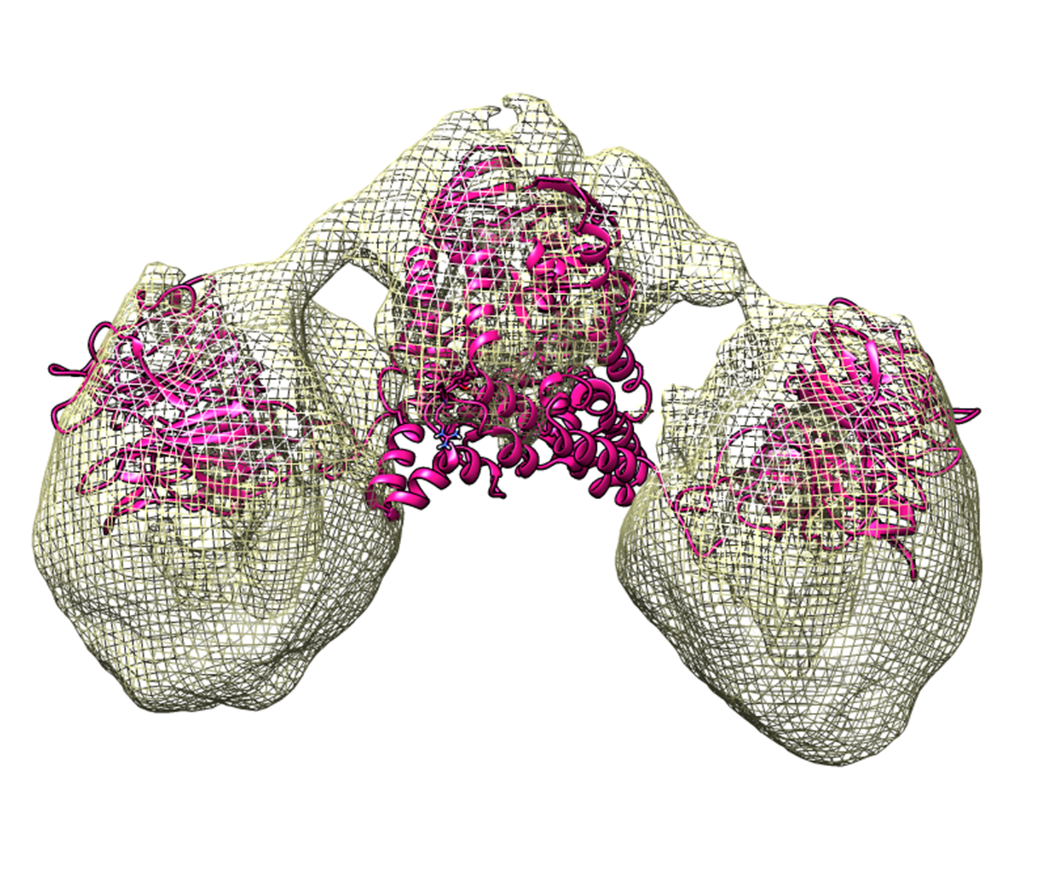

Supplement: Supplementary file 1 — Supplemental figures show the results (Ramachandran Plots) of energy minimization of human Keap1 dimer model (Fig 1S) and the superimposition of the full atom model with the low resolution (24 Å) structure of murine Keap1 dimer (Fig 2S). Fig. 1 S. Ramachandran-plots of Keap-1 dimer. A) Plot before energy minimization. B) Plot after energy minimization. Energy minimization (rigid body and all-atom) was carried out with the protein optimization task of Schrodinger © Maestro using the VSGB 2.0 solvation model. Fig.2S. Superimposition of Keap1 dimer model with electron microscopy reconstruction map of mice Keap1 dimer. Mesh surface corresponds to volumetric electron microscopy reconstruction map (24 Å) of murine Keap1 dimer, magenta cartoon corresponds to all-atom model of human Keap1 dimer obtained with Schrodinger © Maestro Advanced Homology modeling task. [file 1420892.f1.docx]
